# Mucoadhesive Multiparticulate Drug Delivery Systems: An Extensive Review of Patents

**DOI:** 10.15171/apb.2019.062

**Published:** 2019-10-24

**Authors:** Someshwar Komati, Suryakanta Swain, Muddana Eswara Bhanoji Rao, Bikash Ranjan Jena, Vishali Dasi

**Affiliations:** ^1^Department of Pharmaceutics, University College of Pharmaceutical Sciences, Palamuru University, Mahaboobnagar, Telangana-509001, India.; ^2^Southern Institute of Medical Sciences, College of Pharmacy, Mangaldas Nagar, Vijyawada Road, Guntur-522 001, Andhra Pradesh, India.; ^3^Department of Pharmaceutics, Roland Institute of Pharmaceutical Sciences, Khodasinghi, Berhampur-760 010, Ganjam, Odisha, India.

**Keywords:** Mucoadhesive materials, Multiparticulate systems, In-vitro and in-vivo methods, Isolated loop technique, Updated patents

## Abstract

Innovations in pharmaceutical research are striving for designing newer drug therapies to eradicate deadly diseases. Strategies for such inventions always flourish with keys and objectives of minimal adverse effects and effective treatment. Recent trends in pharmaceutical technology specify that mucoadhesive drug delivery system is particularly appropriate than oral control release, for getting local systematic delivery of drugs in GIT for an extended interval of time at a predetermined rate. However, it is somehow expensive and unpleasant sensation for some patients, but still it is needful for getting short enzymatic activity, simple administration without pain and evasion of fast pass metabolism. Usually the vehicles employed in drug delivery of mucoadhesive system have a significant impact that draws further attention to potential benefits like improved bioavailability of therapeutic agents, extensive drug residence time at the site of administration and a comparatively faster drug uptake into the systemic circulation. The drug release from mucoadhesive multiparticulates is contingent on several types of factors comprising carrier need to produce the multiparticles and quantity of medication drug contained in them. Mucoadhesion is characterized by selected theories and mechanisms. Various strategies emergent in mucoadhesive multiparticulate drug delivery system (MMDDS) by in-vitro as well as ex-vivo description and characterization are also critically discussed. Apart from these, the primary focus during this review is to highlight current patents, clinical status, and regulatory policy for enhancement of mucoadhesive multi-particulate drug delivery system in the present scenario.

## Introduction

The modern pharmaceutical developments endeavor towards the redemption of new drug molecules, and therapies by incessant effort of the research scientists in order to eradicate harmful diseases from the civilization. Strategies for such inventions always thrive with the key objectives of minimal adverse effects and effective treatment. The need of desired therapeutic effect of the drug had leads in the development of new controlled release systems. In addition to these, mucoadhesive multiparticulate system (MMS) has accomplished remarkable interest due to their plentiful benefit s as well as short risk of dosage clearance, particular site-specific and regulating efficient plasma drug concentration devoid of improved operation.^1^ Mucoadhesion or bioadhesion is defined as “the state in which two materials are adhered together, which implies attachment of a drug carrier system to a specific biological location”. Adherence of the two materials is attained by contact between a pressure-sensitive adhesive and a surface (mucus membrane).^2^ These two surfaces are held together during the treatment period governed by different forces, which are later explained in theories of mucoadhesion section.^3^ Pharmaceutical technologists often encounter potential challenges for designing new MMS like controlled release oral systems. For obtaining the best possible therapy, this scheme is in capable of implementing a remedy at a pharmacologically better curative efficient rate to an enviable location for the essential stage. During MMS development, several strategies have been employed for site-specific mucoadhesion so as to provide improved and reproducible pharmacokinetics behavior. Unlike conventional formulations, they are less dependent on the gastric emptying, resulting in less inter and intra-subject variability in GI (gastrointestinal) transit time. They are also better distributed and less likely to cause local irritation. In order to renovate the transport time of gastro-intestinal tract, the medicament liberate system originated can be moved to a specific target site or to the absorption site of region which is found at particular site for extended period of time to exploit the corresponding drug dosage release.^4,5^ MMS does not allow dose dumping and possess uncompromising drug safety than the conventional dosage forms. Definitely, it implies to release the whole dosage into the stomach by such phenomenon that leading to pain and ulcer as well as condensed efficacy, because of an enteric-coated tablet having its film coating layer is completely altered and destroyed. On the other hand, in mucoadhesive delivery system, every particulate (single subunit) is made-up with the release characteristics and any smash up only relates to the release behavior of subunit that has been concerned, which ultimately shows a tiny fraction of whole dose. Hence, it is leading to complete decrease of safety related troubles.^6^

## Mechanism ofmucoadhesion

Usually four categories of bio-adhesion have been notable within the biological process. They are commonly stated as a unification of (i) a normal cell to a further normal cell; (ii) a standard common cell to a pathological cell (iii) a cell to a foreign matter; and (iv) an bonding agent to biological substances. In case of mucoadhesion, the foremost phase includes a friendly get in touch with a muco-adhesive material as well as mucus or a biological membrane by each, because of a superior wetting or swelling of the bio-adhesive. The overall mechanism basically includes creation of mucoadhesive bond. Broadly the stages of mucoadhesion composed of two important stages i.e. contact stage (primary) and consolidation stage (secondary).

### Step I (contact stage)

It involves the wetting and consequently swelling of the bioadhesive or polymer which takes place when a polymer is placed on the mucous membrane and results in to a deep contact. Here polymer swelling arises since the substances of polymer have an attraction for water.^7^

### Step II (polymer chains and mucosal membrane Interpenetration)

Just like that in the second phase, the polymer chains of mucoadhesive and the mucosal layer can interact and entangles by formation of adhesive bonds. Later on the contact has been recognized and perforation of the bioadhesive into the crevices of tissue exterior portion. Afterwards a correlation exists and bioadhesive chains impregnate with those of mucus. This phenomenon also had been occurred by force of bonds which rely on extent of perforation among two polymer groups.^8^

### Step III (bonds creation among the entwined chains)

**Figure 1:**
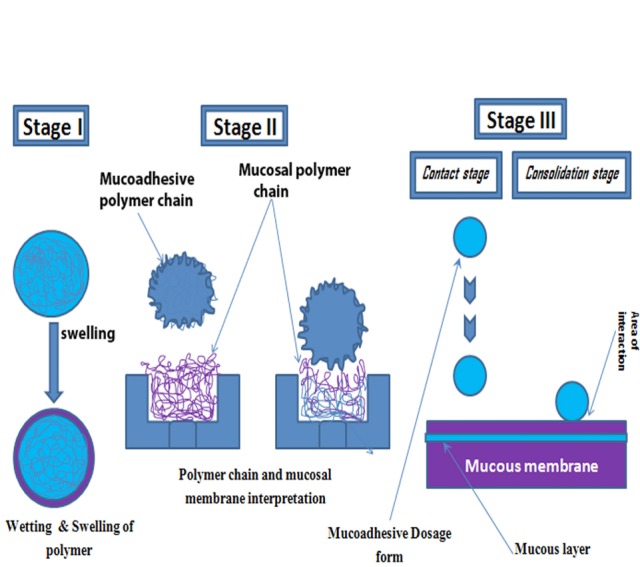
Steps involved in mechanism of mucoadhesion.

Here both collectively recognized as consolidation stage. In this case, the weak chemical bonds can resolve at that time which was depicted in Figure 1. Other types of bond comprise covalent bonds and secondary interactions like hydrogen bonds as well as Vander Waals bonds.^9^

## Factors affectingmucoadhesion

- *Polymer related factors:* Several properties or characteristics of the active polymer play a vital role in mucoadhesion. Among them, polymer molecular weight, concentration, swelling, of polymer chains flexibility, and particular confirmation which may affect the mucoadhesion.
- *Environment associated factors:* pH of the polymer-substrate interface, functional strength and first contact time is able to influence the mucoadhesion.
- *Physiological factors:* Disease state and mucin turn over are the important physiological factors, which can also affect mucoadhesion.^10^

## Mucoadhesiontheories

Mucoadhesion will be able to outline and it is concerned with molecular interactions. The interaction between two molecules is composed of repulsion as well as attraction. The attractive interface would be superior to that of non-specific repulsion. The appropriate occurrence of mucoadhesion, these diverse forces of interactions is entirely narrated by the subsequent theories.

### Electronic theory

Electronic hypothesis concerned to the principle that jointly mucoadhesive and biological materials acquire divergent electrical charges, thus when both resources make contact with, each other, then they swap over electrons foremost to construct a twofold electronic layer at the boundary, where the striking forces within this electronic twofold layer, found out the mucoadhesive potency.

### Adsorption theory

As stated by the adsorption theory, the mucoadhesive machine coheres to the mucus by means of secondary chemical interactions, for example in Vander Waals forces and electrostatic attraction hydrogen bonds, or by means of hydrophobic interactions.

### Wetting theory

The wetting theory implies to liquid systems which related to the current affinity to the surface in order to broadcast over it. Contact angle which is considered as one of the prime measurement tools for the creation of such kind of affinities. The universal rule indicates that the greater affinity correlates to lower the contact angle. The contact angle is supposed to be the identical or close up to zero in order to afford sufficient spreadability.

### Diffusion theory

Diffusion theory narrated to the inter-perforation together of mucin as well as chains of polymer up to an adequate depth in order to build up a semi-permanent adhesive bond. Such a penetration rate absolutely be contingent on the several parameters such as nature of the mucoadhesive chains, diffusion coefficient, flexibility, motility in association with contact time.

### Fracture theory

This is probably one of prime well-known theory in studies, associated to the mucoadhesion measurement by mechanical processes. Once complete formation of adhesion, it totally examine the force required to take apart both thesurfaces.^11^

### Mechanical theory

By proper packing of the irregularities upon a mucoadhesive liquid coarse surface that finally taken as one of the important factor which leads to consideration of adhesion phenomenon by mechanical concepts. In addition to this, such coarseness or roughness steadily grows the interfacial area that’s obtainable for interactions by the subsequent addition of squandering energy and it will be take into account of most significant observable fact of the procedure.^12^

### Mucosal docked vesicle theory

This theory implies about at specific mucosal epithelium vital absorption merely takes place. It may probable that the globules simply can interrelate with the mucous as well as mucosal basal membrane exclusively. Pharmacologically active drugs secluded, in the vesicle that may be liable to spread transversely to the basal membrane of mucosal layer and come into the blood stream for effective distribution at the time of occurrence of docking or releasing.^13^

## Mucoadhesive materials

A supreme mucoadhesive substance should exhibit the capability to integrate jointly hydrophilic and lipophilic drugs. They must illustrate properties of mucoadhesive in its both liquids as well as solid forms. They supposed to be restraining the enzymes of local region otherwise to elevate absorption and should own definite molecular weight and chain length. They should be restricted for meticulous cellular site. They should have an extensive safety range and also they induces endocytosis. Bioadhesive materials are invoked just because of absorption promotes for several routes of administration. Considering the polymers discovery in the earlier year and the later year (20thcentury), they are broadly divided into two categories: (i) First generation or earlier generation mucoadhesive materials and (ii) Second generations or novel mucoadhesive materials.

### First generation mucoadhesive materials

The first generation mucoadhesive substances are often natural molecules or sometimes of synthetic hydrophilic substances enclosing abundant organic functions (carboxyl, hydroxyl and amino groups) that generate hydrogen bonds, which do not stick on especially onto numerous surfaces. Denture fixers were found to be the foremost utility of mucoadhesive materials and the well-known specimens are alginates, chitosans, derivatives of cellulose and carbomers. These are broadly categorized into three types.

- *Cationic:* The Cationic molecules will be capable of interrelate with the surface of mucous, because of its charged which is negative at physiological pH. Mucoadhesion takes place by electrostatic interactions of mucin, containing sialic category along with the amino category in layer of mucous.
- *Anionic:* The polymers of synthetic variety have been derived from poly acrylic acid (Carbomer) which are not only mucoadhesive but also it bears negative charge. In such conditions the physical-chemical yields and parameters (just as instance: van der Waals and hydrogen bonds, hydrophobic interactions,) plays a vital role in the configuration of mucoadhesion that are regulated by the ionic composition as well as pH. One of the broad utility of mucoadhesive systems are polyacrylic acid hydrogels.
- *Non-ionic polymers:* Non-ionic polymers include hydroxyl propyl-methyl cellulose, hydroxyethyl cellulose, and methyl cellulose, a current weaker force of mucoadhesion compared to anionic polymers.^14^

### Second generation materials

New mucoadhesive systems composed of multifunctional materials. They are an alternative to non-specific bioadhesives since they stick on to definite chemical structures upon the surface of mucous or cell. Generally proteins of fimbrial types, lectins, invasions as well as which are acquired by the thiol groups containing cations are to identify, the molecules are some of the instance of these substances.^15^

## Multiparticulatemucoadhesive formulations

During a chosen personification elucidated by mode of case in point, availability and effective delivery of gas emergent components along with numeral of film resembling make a heap of distinguishing particles, which comprise of a mucoadhesive; strong material containing layer as well as interlining layer, regulating the instructions of active material liberate. These active substances which are being placed inside an enclosure of polymer, that is contrasting to gastric juice but it leads to permeability to the intestinal juice-resistant tool. These also composed of minimum one active moiety having mucoadhesive properties in the form of a multiparticulate groundworks. These also contain a blowing agent that get in touch with liquid forms gas. Discrete particles shield to stay behind by means of inclusion of polymers (having, solubility in intestinal juice but resistance to gastric juice) as in the subsequent trend.^16^

- For the formation, the polymer enclosure compartments needs movement of polymer materials in a web form to the molding board that is in association with bores, by utilizing a vacuum.
- Subsequently packing and proper fulfillment in the active material including preparation and also formation of blowing agents.
- Replacement of a subsequent polymer web, as well as locking of compartments by implementing with pressure and high temperature.
- Sorting out and isolate the individual machinery by cutting as well as punching.

## Designs of differentmultiparticulatesystems

Integration of a prevailing drug into a novel drug delivery system can extensively progress its implementation in terms of effectiveness, protection and enhanced compliance of patient. Upon the outline of a novel drug delivery system, usually an existing drug molecule is able to get a novel life, as a result of that it enhances its market worth as well as competitiveness. Consequently, it also expands the patent life of the resultant molecule. Types of mucoadhesive formulations used for different delivery of drugs are enlisted in Table 1.^17-28^

**Table 1:** Types of mucoadhesive formulations used for delivery of drugs

**Type of dosages form**	**Drug used**	**References**
Double layered mucoadhesive tablets	Nystatin	17
Mucoadhesive microcapsules	Glipizide	18
Buccal liposomal delivery	Silymarin	19
mucoadhesive tablets	Naltrexone	20
Chemically modified beta-cyclodextrin complexes	Omeprazole	21
**Type of mucoadhesive formulations used for rectal delivery of drugs**		
Controlled release of solid-reserved micellar solution and suppositories	Metoclopramide HCl	22
Tropical delivery on inflammatory bowel disease.	Amino salicylates and Budesonide	23
**Type of mucoadhesive formulations used for nasal delivery of drugs**		
Microparticulates	Hyaluronan	24
Organogel components intra nasal delivery	Propranolol hydrochloride	25
Nasal administration of chitosan based microspheres	Carbamazepine	26
Transdermal ionophoretic delivery	Sumatriptan succinate	27
Drug transferon all parts of the human nasal epithelial cell monolayer	Fexofenadine hydrochloride	28
**Type of mucoadhesive formulations used for ocular delivery of drugs**		
Occular drug delivery specifying theretina as well as epithelium of retinal pigment.	Polyactide nanoparticle	29
Chitosan nanoparticles as new ocular drug delivery systems.	Chitosan nanoparticle	30

### Diffucaps

In such a category of multiparticulate system the profiles of medicine are subsequently enhanced by casing a lively drug upon a neutral core for instance spheres of sugar, crystals, or granules which in course being followed by the implementation of a rate scheming, competent membrane which is effective. The materials needed for coating either may be water-soluble or insoluble, sensitive to pH, which in turn subject to the needs of individual. Generally, diffucaps beads having a minute in size, very nearly around diameter size of 1 mm, which are packed into a capsule in order to generate the ultimate dosage form. Beads of contradictory release profiles of medicine can also be eagerly united in a solitary or single capsule giving elevated levels of control over release profiles. Diffucaps beads of diverse medicine are united to make suitable solitary dosage units for therapies of mixture or combination remedies.^29^

### Multiparticulatedrug dispersing shuttle

It usually composed of a tablet carrier for the liberation of controlled release beads or pellets in the route of the G.I.T, which maintains the nobility of beads, as well as release characteristics. The disintegration processes carried out in the stomach of the analogous stable tablet carrier so that beads are also uniformly distributed. Due to super disintegration of the tablets, drug discharge from beads is observed. The obtained beads can be further relocated and undergoes formulation to generate a zero order as well as first order release.^30^

### Macrocap

Macrocap composed of immediate release beads prepared by several techniques such as spheronization, pelletization, extrusion etc, or by means of solutions layering or overspreading powders upon seeds of nonpareil. Specifically, the polymers of release modulating be sprayed on the beads by means of several coating techniques. The beads which are undergone coating are accomplished to capsule cells of hard gelatin. Due to proper diffusion related to either osmosis or bioerosion through the membrane of surface that results into the effective release of drugs. The release phenomenon can be either depends upon pH or its activation.^31^

### Orbexa

Orbexa technology is a specific type of multiparticulate system that facilitates loading of huge medicament which is solely appropriate for products which need granulation. This device brings out beads that are in control of size as well as density by means of miscellaneous techniques such as granulation, extrusion and spheronization etc. This procedure is only one of its kind, that specific and includes advanced drug loading than others. Some of the prime merits include enzyme sensitivity and flexibility.^31,32^

## Methods of evaluation and optimalcharacterisationof MMDDS

### In-vitro, ex-vivo and in vivo methods

#### Tests measuring mucoadhesive strength

The mucoadhesive strength assessment, with the aim that the force necessary to smash the binding between the mucoadhesive and model membrane. Based upon the direction wherein the mucoadhesive is isolated as from the substrate molecule, it is feasible to break tensile strengths and also to get the objectivity, shear etc. The driving force most often evaluated in such test in order to split the tensile strength. Normally the device can be useful in texture analyzer calculated which might be a disc encompass mucin, a portion of animal mucous from rats. Upon the surface by formation of tensile peak force leads to deformation that results failure, this technique is more commonly utilized in order to analyze solid phase process such as microspheres. Analysis of tests and interactions of molecules that concerned in the principle of mucoadhesion. The advanced utility of low down frequency dielectric spectroscopy indicates an effort to learn interactions arises between the molecules as well as glycoprotein’s to the mucous at the point of interface, of a particular gel-mucus, which ultimately communicate proper learning of response of materials. This is employed in case of a field associated to electricity. Normally the electrical energy (voltage) of sinusoidal is employed right through the test sample and subsequently the reciprocation is estimated as a function of the frequency. Due to the competent feedback the absolute permittivity of test specimen is achieved as well as the charge’s possessions altering the system can also be found out significantly. This system can afford instruction regarding the affinity amongst the mucoadhesive system and mucus right through in depth assessment of charged particles motion. This compatibility is obtained successfully based on the ease, by way of that, the particles cross the barrier amongst the mucous membrane and gel separately and the interface between them, an additional system being implemented to find out molecular reciprocal action be the resonant mirror biosensor or it’s be the optical biosensor method. This method is broadly employed for measurement of interaction among different polymers and mucous glycoproteins. It also involves the proper regulation of any interaction takes place among two indefinite molecules in actual time, because of one of them could be incapable and ineffective with non-covalent as well as covalent bonds upon the surface of systems or in solution at the surfaces.^33^ At present, certain optimize in-vivo and ex-vivo techniques of mucoadhesive formulations have been developed by using quality by design approach which is cost effective and even more convenient than other techniques.^34-37^

#### Falling liquid film method

**Figure 2:**
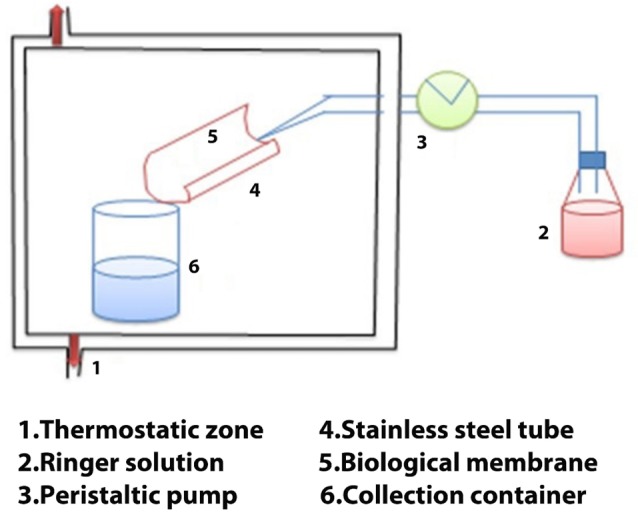
Schematic diagram of falling liquid film method.

In this technique, within a cylindrical cell, the selected mucous membrane is located by means of a temperature regulated at 37°C. First of all, an isotonic solution is moved by using suction right through the mucous membrane and it is accumulated in a beaker (Figure 2). Afterwards, for the particulate system, with the assist of a coulter counter the quantity leftover in the mucous membrane might be added up. The mucoadhesive those have not adhered can be simply evaluated and quantified by means of kigh performance liquid chromatography for semisolid. In this subsequent case, specifically jejunum from rabbits as well as intestinal, porcine stomach, buccal mucous was examined effectively. This technique virtually elucidates clear-cut revelation and improvement in mesophase of liquid-crystalline ahead mucous membrane following the stream of fluids as well as exploration by polarized light microscopy.^38^

#### Imaging methods

Optical microscopes present inadequate isolation and clarity for the study of effects and its impact at a molecular level. This examination intended for both micro as well as nanometer level is highly essential. Ecological circumstances in association with the test or sample are required to be performed that are far-off commencing the biological study of physiology and the surroundings rather than electron microscopy (EM) which elucidates a better appearance. Atomic force microscopy is a moderately novel practice that prevails over numerous boundaries, since it may be used underneath each ecological environment such as in liquids, air or vacuum. It became larger even extra than 10^9^ folds, that permits then vision and revelation of remote atoms which provides a three-dimensional picture of surface.^39^

#### Swelling index

Novel buccoadhesive systems (NBASs) were individually weighed (W0) and located discretely in Petri dishes holding up the phosphate buffer solution of around 4 ml. At common time duration, the NBASs were detached from the Petri dishes and surplus surface water was separated cautiously by means of the filter paper.^13,39,40^ The inflamed or swollen NBASs after that reweighed (Wt) and swelling index (SI) was computed by means of the subsequent formula:

$$SI=\frac{W_{2}-W_{0}}{W_{0}}$$

### In-vivo evaluation methods

The majority of common in vivo techniques to watch bio-adhesion includes:

#### Use of radioisotopes

To find out the consequence of the mucoadhesive of polymers on GI transit time barium sulfate was used as a marker and which is encapsulated in mucoadhesive tablets. By means of automatic faeces compilation machine for collecting the faeces as well as by x-ray examination that supply a non-invasive technique which devoid of affecting the normal GI motility, but it can be monitor total GI residence time. Mucoadhesive predominantly labelled with isotopes such as iodine-123(I-123) or technitium-99, (Tc-99m), as well as chromium-51(Cr-51), indium-113(In-113m), etc., which are being utilized in order to study the shipment or transit of the tablets specifically in the region of Gastro intestinal tract.^41,42^

#### Use of pharmacoscintigraphy

Information on instant and amount of drug release in different distinct of the GI can be obtained. Clinical proof that drug delivery occurs as intended can be available at an early stage. Effect of food on drug-formulation behavior can be determined. Data can be obtained for regulatory authorities showing that products behave in vivo as claimed. Problems arising from the difficulty of prediction of dosage-form behavior based on results of studies related to dissolution test carried out by in-vitro can be overcome. Inter-subject variability in pharmacokinetics profiles will be able to be derived based upon of results of pharmacoscintigraphic evaluations.^43^

#### Use of electron paramagnetic resonance oximetry

Electron paramagnetic resonance spectroscopy is employed in a range of streams of science, for instance, chemistry, physics, biology, for the finding and recognition of free radicals as well as centers of paramagnetic theories for example F-centers. Just as while ice (solid H_2_O) is decaying by revelation to emission, radicals for example, H, OH, and HO_2_ are formed. Just as radicals might be able to be defined and well explained by EPR. Especially both inorganic as well as Organic radicals will be capable of detecting the substances in electrochemical cells that are revealed to illumination by ultraviolet. EPR is typically employed to give the instructions upon a geometry of radical’s as well as the orbital of the unpaired electron in a lot of cases, whereas in further cases the reactions has been arise to make the radicals and their succeeding reactions which are considered as utmost important. Usually in case of archaeological and geological science the EPR/ESR spectroscopy is utilized and considered as dating devices. It also might be implemented to an extensive variety of resources for example, sulfates, phosphates, carbonates, as well as silica or other silicates.^44^

#### Isolated loop technique

Application of an over dosage of barbiturates, the male Wistar rats having an average weight around 300 g, are knocked out and killed by the application of anesthesia. Moreover the small intestine is detached and placed in a solution of 500 mL saline and it is cleaned off properly with physiological saline by the help of a needle of around 5–10 mL/min anticipated for 10 min, after that 20-30 mL/min intended for around 20 min by way of a heating stipulation of 15°C awaiting for extra use. A requisite quantity of microspheres is dispersed in a physiological saline and sonication was carried out accordingly. The suspension of the microsphere is packed into around 15cm in length of small intestine and it is potted properly. Incubation was carried out for the tubes within saline intended for 60 min at 37°C. By the application of the Coulter Counter technique the number of microspheres available in the suspension is counted previous to and subsequent to the adhesion revision. Similarly, by means of counts difference, the proportion of microspheres stick fast toward the tissue is being estimated.^41^

#### Optimal characterization techniques in mucoadhesivemultiparticulatedrug delivery system

The finest approach to assess bioadhesive microspheres is to evaluate the bioadhesive polymer efficiency to extend the GI transit time of the drug at the absorption site thereby raising the absorption rate and bioavailability. The majority of general and appropriate methods to review the mucoadhesive properties of applicant formulations include the in vitro evaluation. These techniques are characteristically significant for evaluating the concept of mucoadhesion through measuring the tensile force that reviews the forces of both attachment as well as detachment. These studies also lead to estimate the shear stress impact and proper measuring the residence time of a formulation bearing bioadhesive upon its mucosal membrane.^45^

## Measurement of tensile stress

### Wilhelmy plate technique

This technique is used conventionally for computation of dimensions of the dynamic contact angles. This makes up of the utilization of a microbalance or a microtensiometer. For the measurement of adhesive microforce, the CAHN dynamic contact angle analyzer (CAHN instruments, model DCA 322, Cerritos) is implemented. The DCA 322 arrangement encompasses an IBM, well-matched CPU and a microbalance configuration.^46^ The device is useful for measuring the bioadhesive force among the mucous tissue and a single microsphere placed on a minute diameter metal wire suspended from the sample loop in microtensiometer.^47^ By the application of CAHN software technique, vital bioadhesive parameters can be analyzed, which are mostly the fracture strength, deformation to failure and the work of adhesion. This system allows the quantity of bioadhesive properties of a candidate material in the exact geometry of the proposed microsphere delivery device and the use of a physiological tissue chamber mimics the in vivo conditions.^48^

### Rheological analysis by Shear stress method

The shear stress is another important technique used to quantify and evaluate the force that produces a mucoadhesive to slide, relating to the mucus layer in a way parallel to their contact plane Test pertaining to adhesion correlates to appropriate measuring the shear stress which includes two figures of glass slides that are being covered by means of mucus film and polymer. Mucus produces a thin film among the two polymers coated slides, and experiment dealings the force necessary to divide the relevant pair surfaces with polymer and a mucous film layer.^49^

### Analysis by electromagnetic force transducer

The electromagnetic force transduceris a powerful device that utilizes the electromagnets which are calibrated in order to take apart a polymer of magnetic loads containing microsphere from a tissue sample. It has the tendency to record the tensile force instructions casually and at a time, as well as more intensification film descriptions of bioadhesive interactions at close to physiological circumstances. This may feasible to execute a precise measure of bioadhesive proportions upon the minute microspheres. The crucial merit of the electromagnetic force transduceris that there will be no physical contact is desirable among the force transducer and the microsphere.

Hence this technique can also be employed successfully to assess the polymer bioadhesion to the definite cell types so that it may be applicable to build up buccal drug delivery system to aim-specified tissues.^50^

## Techniques used for measuring adhesive strength

Several useful techniques are employed for the measurement of adhesive strength such as falling liquid film method, adhesion number and everted sac technique etc.

### Falling liquid film method

It is one of the novel quantitative technique, which deals with the selection of excised section of an intestine which has to be slashed along the lengthways direction upon a plastic flute which is located in an inclined mode. The details of the method have been already discussed.^51^

### Adhesion number

Adhesion number can be stated as the proportion of the quantity of particles adhere to the substrate to the total number of applied particles that are specified as a percentage. Adhesion number improvement is directly interrelated to subsequent raise of adhesion strength.^52^

### Everted sac technique

The everted intestinal sac process is a passive evaluation technique which is associated with the microspheres of polymer and a segment of the everted tissue of the intestine. It is carried out by using a segment of intestinal tissue which has been gathered from rat, everted, ligated at the split ends and packed up by means of saline. It is then inserted into a pipe including a recognized quantity of the saline and microspheres, and agitation was carried out whilst it has undergone incubation for 30mins. The required quantity of sac is thereafter separated and washing and lyophilization of the microspheres were performed. Ultimately the fusion of bind to the sac in percentage is estimated as of weight differentiation of the remaining spheres from the actual microspheres weight. The microspheres that subjected to testing does not need any external strength which is one of the advantage of this method. By means of the everted intestinal tissue at random mode, the microspheres are liberally balanced in solution of buffer and prepared to get in combination. Simultaneously the CAHN as well as everted intestinal sac technique forecast the bioadhesion force in an incredibly analogous mode. Any one of the single technique may be utilized in confidential way for effective scanning of multiple types of bioadhesive polymer. For this reason there is a mutual co-relation and mechanism can exist among the two in-vitro bioadhesion techniques.^53^

## Clinical status of mucoadhesivemultiparticulatesystem

The predefined objective about bioadhesion and small particulates plays a challenging and significant task for the clinical evaluation point of view. A bioadhesive polymer, such as chitosan having properties associated with as an adjuvant, has examined effectively. Its evaluation was carried out in quite a lot of clinical researches, and also in the case of starch microparticles.^54-56^ Only some studies including chitosan systems have been examined in human volunteers. In spite of recurrent studies associated with chitosan based vaccination in the mucous layer in animal designs. For immunization purposes of intranasal therapy, chitosan was effectively utilized to obtain better feedback of antibodies adjacent to a number of proteins, influenza, antigens related to diphtheria, as well as meningococcal conjugates (group-C type) in the case of clinical trials research.^57^ Presently, for the safety, efficacy and protection of novo virus Ligocyte Pharmaceuticals Inc. used the ChiSys® intended for vaccine containing intranasal powder. In- depth clinical trial research revealed about the vaccines that elicit an immunological response and usually able to be tolerated in human volunteers.^58^ It has been found out that, when clinical studies related to any diphtheria toxin, for example: to CRM197 which is a non-toxic mutant, can exhibit the responses of serum antibody by mutual combination with chitosan. This procedure has occurred after successful immunization of intranasal therapy. This concept is virtually equivalent to the theme of proactive immunity.^56-58^ In the present scenario, instead of infants, approximately all the clinical research of intra nasal route is successfully performed by using healthy adult volunteers. By mixing the protocols of immunization through several routes, systematic immunological effect can be generated based on a preclinical point of view. This immunization procedure also includes optimized formulation containing more adjuvants.^59^ The toxin associated adjuvants of mucous like cholera toxin as well as B-subunit of cholera toxin are a major impact. When these are applied through the nasal route they directly connected towards olfactory bulb in central nervous system.^60^ Now a day’s LTK63 which is an adjuvanticity of the mutant of LT in association with Bell’s palsy disorders are needful to evaluate the clinical trials in human beings.^61^

## X-ray photoelectron spectroscopy for analysis of the surface of biomaterials

The analysis of polymer surfaces, its distinctiveness quantitatively through X-ray photoelectron spectroscopy has been leading an active area of research and development. To study the surface analysis of the bioadhesive polymer, it is essential to finding probable interfacial bonding among non-natural or artificial mucus with that of the polymer under exploration in vitro. This will be also needful to recognize the breakdown spot of bioadhesive bond. The concept of Photoelectron Spectroscopy is specified by the scientist Miller and Peppa’s. The GI transit time or residence time of mucoadhesives at the site of administration affords the quantitative information upon their mucoadhesive dimension and its characteristics. The GI residence time of more bioadhesive foundation has been scrutinized by means of fluorescent labeling process and radioisotopes. The dealings of a biomaterial with its atmosphere take place first and foremost at the biotic interface. Bulk characteristics are a vital parameter for judgment of common material properties. The various bulk properties includes heat capacity, tensile strength, etc, as well as a lot of interactions particularly, in relation to the surface area e.g. adhesion, wetting, several reactions of catalytic function, compatibility of blood are the leading factors that plays a significant role for the surface analysis of biomaterials.^62^ Apart from these, now a day’s further vital techniques such as scanning electron microscopy (SEM), EM, and scanning tunneling microscopy are employed for the study, investigation and citations of surface characteristics of microspheres, any alternation formed by means of degradation of the polymer. For effective management of upper GIT infectivity, newly metronidazole loaded floating-mucoadhesive microspheres have developed for the release of drug at a predetermined rate to the gastric mucosal layer. These microspheres are mostly distinguished by SEM for evaluation of targeted drug delivery.^63^ Usually to review and analyses the morphology of surface and bioadhesive characteristics the samples of microspheres are subjected for lyophilization and can be examined under SEM at 150X and 1000X. The microsphere surface texture analysis can be examined by either smooth degradation or coarser texture surface outcomes to either weaker bioadhesive characteristics or stronger interactions mechanically. Any alternation in surface morphology takes place because of the polymers hydrolytic degradation. Usually, the polyanhydrides may be investigated subsequent incubation of the microspheres in the PBS buffer intended for diverse time intervals.^63^

## Analysis of GI transit time by using radio-opaque microspheres

It is an effortless process concerning the utilization of markers that contain radio-opaque, such as barium sulfate (BaSO_4_), which is being encapsulated in microspheres of bioadhesive to conclude the special effects of bioadhesive polymers on GI transit time. The Collection of faeces (by robotic faeces gathering apparatus), as well as inspection by X-ray, produces a non-invasive technique of controlling the whole residence time of GI tract, devoid of usual gastrointestinal motility. Especially mucoadhesives labeled with, Tc-99m, Cr-51, I-123, In-113m, etc are employed to review microspheres transit time in GI tract.^64^

## Feasibility of using mucoadhesive formulation for pediatric population and other injurious diseases

Due to the smoothness of operation and simplicity of application, mucosal vaccines are reviewed as striking approach to fight the promising and re-emerging contagious diseases. Furthermore, mucosal vaccine application affords a number of merits such as it is quite simple and it need not require any class expert persons. It does not engross the utilization of needles, that can considerably result in enhanced vaccine implementation and more prominently avoids the difficulty of blood transmissible infections in rising countries owing to reprocess of needles.^65^ First and foremost in case of pediatrics, kids getting numerous vaccines and vaccination dosages at the age of the first year. This delivery occurred by means of mucosal route that could move away from continual needle injections and the consequential distress intended for jointly kids and their parents. Mucosal vaccination may also result into minor side effects and if the formulation was done rightfully. Then it might possibly be delivered by parents to their kids, leading to avoidance of frequent visits to pediatricians in distinction with vaccines containing injectable.^66,67^ For support the magnification of mucosal vaccination of pediatric population quite a lot of preclinical researches in near the beginning stage of life has been carried out.^68-70^ Moreover the clinical trials conducted in children with an interracial live influenza vaccine have confirmed to be secure and effective, with improved usefulness shown over the injectable standpoint.^71-74^ A number of deadly injurious diseases can be readily cured by using MMDDS. Some of the chosen examples include treatment of recurrent aphthous stomatitis.^75^ This is one of the unceasing syndromes of the oral cavity. Recent discovery states the origin of oral paste formulation of Triamcinolone Acetonide which has an effective drug release system for the curing of recurrent aphthous stomatitis.^75^ Similarly mucoadhesive buccal discs and sublingual films of sumatriptan-metoclopramide combinations are readily been employed for treating adjunctive therapy, which has an enhanced bioavailability.^76^

## Recent patents

Recent patents on mucoadhesive multiparticulate drug delivery systems are depicted in (Table 2). The strategies of recent patents are described as follows;

**Table 2:** Recent patents on mucoadhesive drug delivery systems

**Patent No**	**Title of the patent**	**Types of delivery systems**	**Major invention**	**Conclusions**
WO/2003/086297	Multi-layer mucoadhesive drug delivery device with bursting release layer	Tablets	Multi-layer mucoadhesive drug delivery system includes: (a) mucoadhesive layer, including polymer of non-ionic, anionic polymer swelling modifier, and at buffering agent; (b) effervescent layer, containing permeation enhancer, effervescent couple, comprising an anhydrous acid and an alkalizing agent, and binder; and, (c) at least one active agents contained in both.	The active agent in the effervescent layer is released from the drug delivery device in association with permeation enhancer within 10 minutes, more nearer to 5 minutes, mostly within 1 minute, to a subject and active agent in the mucoadhesive layer is released in excess of a stageof at slightest 8 hours, extra at 12 hours, mostly at 24 hours, follows zero-order kinetics.
US20110028431	Oral mucoadhesive dosage form	Tablets	It includes a mixture of a polymeric solubility enhancer, which is non-ionic a polymer, of mucoadhesive filler, a disintegrant, and a pharmaceutically active mediator, composite of Cannabinoid-cyclodextrin showing an enhanced property chosen from enhanced steadiness, superior yield of product and superior homogeny of product	The resultant complexes might be united with agents related to de-complexion phenomenon and/or spread upon a substance carried matrix composed of a hydrogel-forming polymer to give improved absorption of the cannabinoids by means of mucosal oral region and decreased intake of the cannabinoids as contrast with recognized accessible cannabinoid-carrying oral pharmaceutical unit dosages.
WO/2006/069911	Mucoadhesive pharmaceutical compositions comprising chemoattractants.	Gels	**T**he innovation related to muco-adhesive pharmaceutical constituents consisting a polymer and a chemo-attractant in which the pH of the constitution is superior than 6 that is helpful in the medication of a anogenital or oral illness, predominantly an anogenital or oral-disorderarised by means ofthe human papillomavirus.	It has been observed that a chemo-attractant extensively improved the chemotaxis of an antigen performing cell and that the addition of a chemo attractant in a muco-adhesive constitution bearing a pH more than 6, stimulated the penetration of an antigen exhibiting cellinto a–transported epithelium of human papilloma virus. As a result, the configuration in accordance with the discovery will be helpful in the management and medication of anogenital as well as oral disorders, principally an anogenital or oral disorder formed by means of human papillomavirus.
US20100100064	Ostomy devices Mucoadhesive	Ostomy appliances	The present invention provides a biocompatible adhesive for securely adhering ostomy appliances simultaneously to the body and the stoma. The ostomy appliance is comprised of an adhesive component and a body waste collector component, wherein the adhesive component includes a mucoadhesive component. The mucoadhesive component comprises a polymer with functional groups that provide adhesion to skin and stomach.	It is a purpose of the current innovation to give a biocompatible adhesive for securely adhering ostomy appliances simultaneously to the body and the stomach.
US8663688	Semi-solid mucoadhesive formulation	Gels	Semisolid muco-adhesive dosage forms specifically meant for vaginal implementation with enhanced organoleptic as well as technical characteristics, which holds not less than two bioadhesive polymers of geland an active pharmaceutical ingredient.	It is needful in the cure and protection and/or medication of a range of pathological disorders in mammalians or animals.
US20140056949	Controlled release mucoadhesive systems	Toothpaste, Mouthwash, Mouth rinse, Gel, Paste, Spray, Chewing-gum, Lozenge.	Controlled Release Mucoadhesive formulations for chemical agents for suppresses of oral cancer and lesions of precancerous cells, as well as the techniques for making the formulations are explained particularly, the innovation associated to gels of bioadhesive bearing a hydrophobic formulation (fenretinide), formulated intended for limited release for the chemical suppression of precancerous wounds as well as oral cancer.	The present invention provides a formulation for transmucosal application that is simple to produce, that shows effective steadiness and permits for formulation pliability and enable for accurate manage upon the dosage applied and the outcome delivered is easy, and suitable for application, effortless in handling and which encourages high patient conformity as well as acceptance.
US8529939	Mucoadhesive drug delivery tools and methods of preparing and utilizing thereof	Wafer, Tablet, Cylinder, Sheet, Particles or Sphere.	The current discovery based on to muco-adhesive drug delivery tools and their techniques of production and usage. More especially the current innovation signifies to muco-adhesive drug delivery machineries consisting one or additional refined biocompatible proteins united with one or additional solvents which are of biocompatible in nature and also includes one or more than one mucoadhesive agents. The mucoadhesive drug delivery tools might include one or additional pharmacologically active agents too.	The medicament release tools of the current discovery stick on to tissue of mucosa, in this manner affording a vehicle for liberation of the pharmacologically active agent(s) in the course of such tissue.
WO/2013/188979	Mucoadhesive nanoparticle delivery system	Injectable Preparations, Ointments, Pastes, Creams, and Gels, Powders and Sprays	The nanoparticles are formed from amphiphilic macromolecules conjugated to a mucosal targeting moiety in such a manner that the surface of the nanoparticle is coated with the targeting moiety. The surface density of the targeting moiety can be tuned for adjustable targeting of the nanoparticles to a mucosal site without substantially compromising the stability of the particles. The particles were found to have high loading efficiency and sustained release properties at the mucosal site. The present disclosure also relates to polymers and macromolecules useful in the preparation of the mucoadhesive nanoparticles, as well as compositions, methods, commercial packages, kits and uses related thereto.	The particles were found to have high loading efficiency and sustained release properties at the mucosal site. The present disclosure also relates to polymers and macromolecules useful in the preparation of the mucoadhesive nanoparticles, as well as compositions, methods, commercial packages, kits and uses related thereto. The nanoparticles can be tuned for controlled targeting and adhesion of the nanoparticles at a mucosal site without substantially compromising the stability of the particles.
US20150174076	Mucoadhesive tools for release of active agents	Wafers	Explained in this are systems and techniques for transmucosal release of active agents. In some personification a system may encompass one or additional mucoadhesive tools designed for release of an active agent.	In a few embodiments, a system may include at least one or additional muco-adhesive tools designed for release of an active agent. In, one feature, muco-adhesive tools forrelease of active agents are afforded. In further cases, the tool might be at least moderately by enclosed and capsulated by means of backing layer containing nominal permeability to the active agent. The tools can in some personification, include a layers plurality.
US20090098203	Mucoadhesive Tetracycline Formulations	Mouth rinse or Tablet	Mucositis is provided and/or cured by applying to a patient a formulation comprising a tetracycline and not less than one polymer bearing cationic groups and/or mucoadhesive substance. The tetracycline might be in the shape of a pharmaceutically suitable either salt or a base. The formulations as an option can also include an agent which is antifungal to protect fungal over development because of decline in the usual oral flora by means of the tetracycline.	Constituents encompass the benefit of long-lasting preservation and tendency of the tetracycline in the mucosal layer of the oral cavity.
US20100144618	Constituents including an trefoil peptide of intestine as well as of a mucoadhesive	Oral spray, Oral rinse, Ointment, Paste, Cream, Gel, Chewing gum, Chewable Tablet, Lozenge, Bioerodable film.	The innovation aspects constituents enclosing an intestinal trefoil peptide of intestine and a mucoadhesive excipient. This types of compounds are needful, e.g., for the medication or cure of lesions. Constituents including an trefoil peptide of intestine and a mucoadhesive excipient might be prepared in grouping with one or additional therapeutic agents.	This invention features a method for treating a lesion of the upper alimentary canal in a mammal by appling to the mammal a pharmacological as well as beneficial conclusion amount of a trefoil peptide. Preferably, the mammal is a human. Treatment or prevention of lesions according to the invention can speed healing, reduce pain, delay or prevent occurrence of the lesion, and inhibit expansion, secondary infection, or other complications of the lesion. Preferably, the mammal is a human. In particularly useful embodiments, the trefoil peptide is SP, pS2, ITF, ITF_15-73_, ITF_21-73_, ITF_1-72_, ITF_15-72_, or ITF_21-72_, and is present in a pharmaceutical composition containing a pharmaceutically acceptable carrier. The trefoil peptide may be administered as a monomer, a dimer, or another multimeric form.
US8703177	Abuse-impervious mucoadhesive tools for release of buprenorphine	Patches	The present creation affords abuse prevention mucoadhesive tools for release of buprenorphine. Each machinery composed off a mucoadhesive layer, usually a backing layer, as well as the pH in every layer is chosen, in such a way that of buprenorphine absorption can be maximized.	The current investigation is based upon, minimum in part, on the detection that opioid agonist bioavailability,for example buprenorphine, liable in the mucoadhesive layer of a bi-layered, abuse-resistant transmucosal drug delivery tool which is not only being pretentious by layer of mucoadhesive pH but is happened by the backing layer pHthat live in upon the lingualsurface of the bi-layer film. It does comprise an opioid which is not agonist just like as naloxone. For that reason, together the pH of the mucoadhesive surface, in accordance with the pH of the backing layer might be selected such that the inclusion of buprenorphine since that mucoadhesive layer looks identical or and superior as that of absorption from the mucoadhesive surface of a tool with an not-buffered backing layer, at the same time as the naloxone being absorbed if exist in the backing layer, is hampered.
WO/2015/126841	Nutritional and therapeutic mucoadhesive formulations	Liquid or Gel	A supplement formulation, comprising a mucoadhesive and an effective amount of one or more of a medicinal food or a nutritional supplement is described as well as use for the delivery of same to mucosal surfaces. The supplement formulation might be in the shape of gel or a liquid.	This disclosure provides mucoadhesive formulations which contain medical foods and/or nutritional supplements in which primary beneficial medical effect is provided by either the medical food/ nutritional supplement or the viscous mucoadhesive formulation. In either case, the other component provides an additional or additive medical effect.
EP2298284	Mucoadhesive pharmaceutical formulations	Suppositories, Emulsions	The innovation based upon to formulations which are having pharmaceutically active ingredients for usage in the application of lipophilic drugs by means of mucosal layers. In exacting the innovation affords constituents of pharmaceutical dosage form intended for usage in application of a lipophilic drug through a surface of mucosal layer that upon hydration shape an emulsion comprising the lipophilic drugs which is competent of attaching to a surface of mucosal layer and permitting controlled release of the drug. The innovation further promotes formulations containing pharmaceutical dosage form that implies, as chief active ingredients, accurate or unification of cannabinoids in pre-defined proportions.	The innovation corresponds to the pharmaceutical formulations for usage in the application of drugs, in meticulous lipophilic substances mean to the medical treatment, through surface of mucosal layer.

Chen and Liang invented mucoadhesive multilayer drug delivery tool through a mechanism of bursting layer liberation or release. In such a drug delivery tool for executing either a single active agent or more than one of active agents to a subject matter are emphasized and afforded. Especially, mucoadhesive multi-layer drug delivery device is afforded in addition with (a) a mucoadhesive layer, enclosing the smallest amount of single polymer of ionic, at least one anionic polymer, at least one swelling modifier, and at least one buffering agent; (b) an effervescent layer, containing at least one permeation enhancer, an effervescent couple, comprising an anhydrous acid and an alkalizing agent, and at least one binder; and, (c) minimum one active agent; while not less than one active agent is contained in both the mucoadhesive layer and the effervescent layer; whereas not less than one active agent is enclosed in the effervescent layer is released from the drug delivery device along with at least one permeation enhancer in 10 minutes, more selectively within 5 minutes, most considerably in 1 minute, of administration of drug delivery device to a subject and wherein the minimum single active agent present in the mucoadhesive layer is released from the drug delivery device over a period of minimum of 8 hours, more selectively minimum 12 hours, most selectively at least 24 hours, preferably with zero-order kinetics. Methods of administering active agents to a subject using such multilayer mucoadhesive drug delivery devices are also provided.^77^

Zerbe and Paiement invented mucoadhesive dosage form by oral origin, that involve the formulation having the direct compression technique appropriate for preparation of sublingual or buccal dosage forms inclusion of a combination containing non-ionic solubility of polymer enhancer, as well as carries a polymer which is made up mucoadhesive, a disintegrant and a filler. In addition to this, it also contains a pharmaceutically active agent. This invention relates to oral pharmaceutical dosage forms and more particularly to oral dosage forms in which a substantial portion of the active ingredient is released in the oral cavity for oral adsorption. This invention also relates to oral pharmaceutical dosage forms and more particularly to buccal and/or sublingual oral dosage forms comprised of at least one pharmaceutically active cannabinoid complexed with cyclodextrin.^78^

Herman et al invented specific pharmaceutical ingredients of mucoadhesive made up of chemoattractants, The creation correlate to the composition enclosing mucoadhesive pharmaceutical which comprises a chemoattractant and a polymer in which the constituents of pH is more than 6 that is needful for the cure of oral as well as anogenital disorders and illness, however the diseases can be formed by human papilloma virus.^79^

Sambasivam invented ostomy devices mucoadhesives and the present invention provides a biocompatible adhesive for securely adhering ostomy appliances simultaneously to the body and the stoma. Invention to provide a biocompatible adhesive for securely adhering ostomy appliances simultaneously to the body and the stoma. In one embodiment, the mucoadhesive is part of the appliance prior to use. In another embodiment, the mucoadhesive is used as a separate component to coat the stomal and persitomal area prior to attachment of the appliance. When the mucoadhesive is part of the appliance, it is continuously or discontinuously distributed in the whole Wafer and/or around the central area of the Wafer contacting the stoma. When the mucoadhesive is a separate component, it can be delivered to the stomal and persitomal area in the shape of a liquid, foam, gel, film, paste, sheet, powder, or a combination thereof.^80^

Acebron et al invented mucoadhesive formulations which in Semi-solid form and the formulations are specified for vaginal function, in accordance with advanced organoleptic and technical uniqueness and character, that exhibit not less than two gelling polymers of bioadhesive types as well as an active pharmaceutical ingredients, needful to overcome and cure in variety of pathological disorders specifically in mammals or animals.^81^

Mallery et alinvented controlled release mucoadhesive systems and the formulations developed for eradication and complete cure by chemical, oral and by precancerous cells, lesions, etc., and several techniques for formulation preparation are emphasized. In a first broad aspect, there is provided herein a formulation, comprising: minimum one mucoadhesive material; minimum one constituents of retinide or a pharmaceutically suitable and granted salt of the thing which mentioned; in accordance not less than one permeation intensifier of transmucosal mediator or agent, which is chosen for enhancing permeation of the retinide composition across a mucosa; and; as an alternative, not less than one solubilizing agent for enhancing release of the configuration of retinide from the mucoadhesive material.^82^

Masters and Berg invented mucoadhesive drug delivery strategy and techniques of making and use. Further exclusively the present invention correlates to the mucoadhesive drug delivery tools consisting of biocompatible decontaminate and pure proteins united through one or extra solvents having biocompatible as well as one or additional mucoadhesive agents. The mucoadhesive drug delivery system can also comprise one or additional active agents of pharmacological response. The drug delivery tools of the present investigation stick onto tissue of mucosal layer, in this manner affording a vehicle for release of the pharmacologically active agent (s) by means of such kind of tissue.^83^

Thoral et alinvented a mucoadhesive sustained-release vaginal tablet comprising at least one probiotic strain of the genus Lactobacillus compressed with an excipient suitable for conferring upon tablet the properties of vaginal wall mucoadhesion and sustained release. The excipient is present in an amount which allows both good adhesion to the mucosa and a sustained release of the strain. The sustained release must meet two requirements to ensure the viability of the strain and enable spaced administration in time of the tablet. It is in fact, seeking a treatment that allows administration of up to one tablet every other day, preferably at most every 3 days or more.^84^

Gu et alinvented mucoadhesive nanoparticle delivery system. The nanoparticles are formed from amphiphilic macromolecules conjugated to a mucosal targeting moiety in such a manner that the surface of the nanoparticle is coated with the targeting moiety. The surface density of the targeting moiety can be tuned for the adjustable targeting of the nanoparticles to a mucosal site without substantially compromising the stability of the particles. The obtained nanoparticles have high loading efficiency and sustained-release properties at the mucosal site.^85^

Harris et al invented mucoadhesive tools intended for delivery of active agents explained this processes and methodology for medication enters through the mucous membrane and release of vital active agents. In some illustration a scheme might include one or additional strategy of mucoadhesive system which arranged for delivery of a dynamic and active agent.^86^

Lawterinvented mucoadhesive tetracycline formulations and mucositis which act towards as well as cured by the administration to a forbearing or victim with a formulation bearing a tetracycline and which having not less than one cationic polymer and/or substance those are mucoadhesive. The tetracycline might be in the shape of a suitable base or a salt and pharmaceutically significant. The formulations also might be include an antifungal agent as an alternative, in order to diminish and overcome the fungal high growth because of decline in the regular oral flora by means of the tetracycline. The formulation might be produced into solid or liquid unit doses like tablet or mouth rinse. Such configuration encompasses the benefit of extended protection of the tetracycline in oral cavity mucosal region.^87^

Podolsky et alinvented the configuration including a trefoil peptide (protein) of the intestine and a mucoadhesive, in accordance with the innovation aspects configurations comprising a trefoil factor peptide of intestinal location and an excipient of mucoadhesive system. This type of substances are beneficial, e.g., for the complete eradication or healing of lesions. By using one or additional therapeutic agents the formulations might be prepared by including constituents of intestinal trefoil peptide and a mucoadhesive excipient or sometimes their combination can also be preferred for consideration. A mucoadhesive excipient can be added to any of the previously described pharmaceutical compositions. The mucoadhesive formulations coat the upper alimentary canal providing protection, inhibiting irritation, and accelerating healing of inflamed or damaged tissue. Mucoadhesive formulations also promote prolonged contact of the trefoil peptide with the mucosal epithelium. Mucoadhesive formulations suitable for use in pharmaceutical preparations delivered by mouth are better recognized and well-known in the art (US 458, 879). Particularly useful mucoadhesives are hydrogels composed of about 0.05%-20% of a water-soluble polymer such as poly-(ethylene oxide), poly-(vinyl alcohol), poly-(ethylene glycol), PVP i.e poly-(vinylpyrrolidoine), poly-(acrylic acid),poly(hydroxy ethyl methacrylate), hydroxyethyl cellulose, hydroxyethyl methyl cellulose, chitosan and all their combinations. These polymeric formulations can also contain a dispersant such as sodium carboxymethyl cellulose (0.5-5. 0%).^88^

Finn and Vasisht invented abuse-impervious to mucoadhesive tool for buprenorphine delivery and innovation is established, at least in fraction, on the innovation that an opioid agonist bioavailability, such as buprenorphine, legally responsible in the mucoadhesive covering of a bilayered, misemploy-resistant transmucosal drug delivery system which is quite much influenced by the mucoadhesive layer pH, as well as being induced by the backing layer pH which ultimately occupies on the lingual portion of the bi-layer film. Such kind of layer may or may not hold an opioid but not agonist or antagonist, however in the chosen personification of the configuration of the corresponding backing layer, it contains an opioid receptor antagonist, for instance as naloxone. Consequently, together the pH of the backing as well as mucoadhesive pH layer may be preferred to facilitate the absorption of the substance e.g., buprenorphine from the layer content of mucoadhesive that is analogous or superior than incorporation from the device containing mucoadhesive layer among non-buffered backing layer, while the naloxone absorption, if current in the backing layer, is obstructed. The current creation is also related to slightest in fraction, on the amazing innovation, so as to the mucoadhesive procedures by means of buffered backing layer might include lesser dosages of naloxone, whereas yet affording abuse prevention. The naloxone dosage supposed to be lowered, in a way that the w/w buprenorphine to naloxone proportion is greater than the proportion of 4:1, granted in the art as being competent for affording abuse avoidance. In a few case of illustration, the proportion of w/w buprenorphine to subsequent naloxone exists in the mucoadhesive appliance of the discovery should be among 4:1 and 10:1. In a particular representation, the proportion of w/w buprenorphine to naloxone is 6:1. This kind of tool is beneficial and much important since it may afford efficient abuse prevention at a lesser dosage of naloxone.^89^

Nowotnik et alinvented nutritional and therapeutic mucoadhesive formulations, for a supplement formulation, comprising a mucoadhesive and an effective amount of one or more of a medicinal food or a nutritional supplement is described as well as use for the delivery of same to mucosal surfaces. The supplement formulation may be located in the shape of a gel or a liquid.^90^

Whittle et alinvented mucoadhesive pharmaceutical formulations and the discovery corresponds to formulations of pharmaceutical dosage form utilized in case of application of lipophilic medicaments throughout the surroundings of mucosal layer. In exacting the innovation affords pharmaceutical configurations utilized in the application of a lipophilic medicament through a surface of mucosa that in the form of hydration, an emulsion comprising a lipophilic medicament that can be able to adhere towards the surface of mucosal layer and permitting controlled release of the drug. The discovery further promotes and gives the formulations inclusion of pharmaceuticals that possesses, as chief active ingredients, of cannabinoids and their definite combinations in established proportions.^91^

Rosario et al invented a multiparticulate system contains oral form in application comprising pellets in the size ranging from 50 to 2500, Sg (mm) which are significantly composed of firstly an interior layer of matrix bearing nanoparticles so as to enclose an active ingredient of nucleic acid and being entrenched in a polymer matrix which shows a mucoadhesive impact. Secondly an outer film coating, considerably comprising of either an anionic polymer or copolymer with the intention of manufactured optionally by means of adjuvants pharmaceutically standard particularly the emollients.^92^

Allan et alinvented a release system that includes nanocomposite mucoadhesive substance such as chitosan that is enclosed in an exterior customized nanoporous nanoparticles of colloid such as silica additional colloid-creating substances principally oxides of metal. Pharmaceutical drug delivery models might be given by attaching a medicament to corresponding silica or chitosan-based nanocomposite exclusively by the addition of a medicament or other active mediator throughout the in-situ gelation of colloidal silica. When pure energetic agent e.g., amoxicillin or other antibiotic mediators are employed than it might be utilized in the management of ulcers in the stomach.^93^

## Conclusion

MMDDSs provide greater adaptability and flexibility to the clinicians or which take part in product growth commanding novel devices to improve the efficiency of treatment. The thought of utilizing bioadhesive resources in connection with surfaces of the mucosal layer, as a technique to progress the effectiveness of therapeutic medications that has been of immense curiosity in the pharmaceutical ground as near the beginning discoveries in the field of mucoadhesion. Studies related to mucoadhesive systems have focused on a wide collection of aspects. Other than single-unit dosage forms the effect can be enhanced better by using multiparticulate drug delivery system since, GI transit time is more reproducible and countable. In case of oral drug delivery, the metabolic function of the G.I tract enables not only the intense barricade but also a demanding concept. The broad inter-intra theme variableness of gastrointestinal residence time is a significant rationale which can have a considerable effect upon the bioavailability of medicine. When the mucoadhesive formulation is anticipated for universal and routine usage, the extensive time interval in which the tool leftover’s in connection with the consequences of absorptive mucosal membrane, in a number of cases, to enhance and raise the bioavailability of drug as well as its effectiveness.

## Current status and future directions

The present study emphasizes the recent position of the formulation aspect, future requirements for novel molecules that intended for targeted drug delivery. In order to enhance the bioavailability of orally incompetent drugs by replacing the design of formulations, the experts and the research scientists have done incessant discoveries to build up the buccal adhesive systems right through a wide variety of access. At present global development, the authors conclude that the ability of the succeeding bio-adhesive polymers of second stage generation is vast, as they have remodeled the thought of mucoadhesion by means of novel findings emerging from fundamental research on these new substances. The delivery of the drug is aimed at buccal mucosa by shielding the local surroundings is also ahead curiosity in case of the innovative buccal adhesive delivery system. Apart from these in merchandise and profitable point of view the flourishing dosage forms, are currently the liquids solids as well as gels those are administered into the oral cavity. The formulations belong to the vaccine and of small proteins or peptides delivery are the epitomes of upcoming trends of the buccal adhesive drug delivery system. Especially the micro-particulate bioadhesive systems are mesmerizing as they provide a defense to therapeutic entities. The improved absorption, result from enlarged contact time afforded by a component of bioadhesive. Exhilarating challenges stay behind to persuade the drug’s bioavailability obliquely the buccal mucosa. Research concerned to the mucoadhesion is a challenging thought and it has versatile applicability. This is an improvement region whose purpose is the extension of new innovative strategy and additional “clever” polymers, as long as the creation of innovative methodologies that can further improve and simplify the thought of mucoadhesion. The formulation of MMDDS can improve and coordinate the passage time throughout the GI tract by means of possible advantage of additional reproducible drug bioavailability. Hence delivery of this system to the colon can be of immense advantages for local management of cancer raised in the colon and several colonic disorders, like inflammatory bowel disease and infections due to Clostridium difficile. However, the union of colonic-drug delivery & mucoadhesive thought has enormous potentiality, to enhance the effectiveness in the healing of colonic diseases in the coming future.

## Conflict of Interest

The authors have no conflict of interest.

## Ethical Issues

Not applicable.
